# The Effect of miR-505-5p on Inhibition of Serum Uromodulin Ameliorates Myocardial Inflammation and Apoptosis Induced by Ischemia-Reperfusion

**DOI:** 10.1155/2022/3521971

**Published:** 2022-10-03

**Authors:** Dongsheng He, Jun Hu, Yuhai Lu, Weikun Jia, Minxue Wei, Xiaofei Zeng, Hong Wang

**Affiliations:** ^1^Department of Cardiothoracic Surgery, First Affiliated Hospital of Chengdu Medical College, Chengdu 610500, China; ^2^Department of Cardiology, First Affiliated Hospital of Chengdu Medical College, Chengdu 610500, China; ^3^Department of Pediatrics, Chengdu Xindu Maternal and Child Health Hospital, Chengdu 610500, China

## Abstract

**Background:**

It has been found that miR-505-5p is closely related to cardiovascular metabolic risk factors. Nonetheless, there is little research analyzing miR-505-5p for its role as well as molecular mechanism in myocardial injury caused by ischemia-reperfusion (I/R).

**Methods:**

This work utilized quantitative reverse transcriptase PCR (qRT-PCR) for detecting miR-505-5p and serum uromodulin (sUmod) levels. sUmod, interleukin-1beta (IL-1*β*), IL-6, IL-10, caspase7, caspase9, tumor necrosis factor-alpha (TNF-*α*), Bax, and Bcl-xL expression was detected by western blot. Bioinformatics database was used for target prediction and miR-505-5's target was determined by luciferase reporter gene assay.

**Results:**

Relative to sham group, sUmod was highly expressed within myocardial I/R injury (MIRI), whereas sUmod silencing significantly decreased the heart weight/body weight ratio, reduced serum myocardial enzymes expression, ameliorated I/R-mediated myocardial apoptosis, and inflammation. TargetScan bioinformatics database and luciferase reporter genes confirmed that sUmod was miR-505-5p's direct target gene, besides, miR-505-5p overexpression significantly improved the myocardial injury score, increased IL-10, decreased TNF-*α*, IL-1*β*, IL-6 expression, decreased caspase7, caspase9, Bax expression, and increased Bcl-xL expression. More importantly, overexpression of sUmod abolished miR-505-5p overexpression's role in I/R-mediated myocardial apoptosis and inflammation.

**Conclusion:**

miR-505-5p can improve I/R-mediated myocardial apoptosis and inflammation by targeting sUmod. In this study, miR-505-5p is related to MIRI pathogenesis, which provides the new possible targeted therapy in patients with MIRI.

## 1. Introduction

Acute myocardial infarction (AMI) represents a common factor leading to disability and mortality in patients globally. In emergency situations, timely thrombolytic treatment or surgical coronary artery bypass grafting (CABG) and direct percutaneous coronary intervention (PPCI) can effectively limit myocardial infarction (MI) size and improve heart function as well as patient prognosis [1]. However, myocardial reperfusion sometimes results in myocardial ischemia-reperfusion (I/R) injury (MIRI). MIRI will reverse certain benefits of blood flow restoration. MIRI has the features of cardiomyocyte edema, myofibrillar rupture, sarcolemmal destruction, and the appearance of calcium and phosphorus granules in mitochondria [2, 3]. Some previous studies have demonstrated that mitochondrial dysfunction [4, 5], oxidative stress [6, 7], intracellular calcium overload [8], inflammation [9, 10], and apoptosis [11] were related to MIRI. Up to date, targets for these harmful mediators aye the focus of treatment to guard against MIRI. However, within the complicated clinical environment, MIRI can not be effectively prevented. Therefore, it is a focus of the current research to find a new treatment for protecting cardiac MIRI.

Serum uromodulin (sUmod), called Tamm-Horsfall protein (THP) as well, represents the glycoprotein that has a 80-90 kDa molecular weight (MW). Its expression can be detected within epithelial cells in Henle ring thick ascending branch (TAL), but less in the early distal convoluted duct [12]. sUmod is a new biomarker for renal activity as well as renal tubular integrity, which is considered to be related to cardiovascular diseases (CVDs) together with the overall mortality among cardiovascular high-risk cases. A study has shown that sUmod is negatively related to CVDs and overall mortality among cases undergoing coronary angiography [13]. sUmod is negatively correlated with several cardiovascular risk factors, including diabetes and arterial hypertension, which may be one of the reasons why sUmod has cardiovascular protective effects [14]. As reported, sUmod significantly decreased within diabetic nephropathy (DN), acute tubular necrosis, as well as inflammatory cytokine-induced hyperprostaglandin E syndrome [15]. Meanwhile, lower sUmod levels were associated with higher cardiac mortality, increased systemic reactive oxygen species, and increased susceptibility to type 2 diabetes with decreased glucose metabolism [16].Moreover, sUmod promoted the proliferation and phagocytic activity of mononuclear macrophages, and the loss of sUmod aggravated the immune response in renal I/R injury [17] .

microRNAs are a small, noncoding family of RNA molecules that have critical effects on cell growth, differentiation together with organ development. miRNAs can directly bind to target mRNA's 3′-untranslated regions (3′-UTR) for regulating gene expression, thereby inducing cleavage or translation inhibition. miRNAs have crucial effects on various inflammatory diseases. miR-505 was initially subject to transcription to the 84-BP pre-mir-505, and later processing to the mature miRNA, which included miR-505-3p and miR-505-5p [18]. miR-505 shows abnormal expression within prostatic cancer (PCa), breast cancer (BC), bladder cancer, colorectal cancer (CRC), and hepatocellular carcinoma (HCC) [19–21]. A study has found that miR-505 decreased HMGB1expression to inhibit autophagy activation of BoDV-1, and ultimately lead to fatal encephalitis in humans [22]. miR-505 was abnormally upregulated in mice with laser-induced choroidal neovascularization, and miR-505 inhibitor inhibited neovascularization and expression of vascular endothelial growth factor (VEGF) [23]. Additionally, miR-505-5p, which is an oncogene, can enhance lung cancer cell growth and inhibit their apoptosis by targeting apoptosis inducing TP53AIP1 [24]. Meanwhile, miR-505-5p also has the tumor suppressive effect on cervical cancer via the target of cyclin dependent kinase 5 [25]. However, it is not clear whether sUmod is miR-505-5p's direct target gene, and whether miR-505-5p can improve I/R-mediated myocardial apoptosis and inflammation by inhibition of sUmod.

This work focused on investigating miR-505-5p's effect on myocardial I/R injury (MIRI) as well as its possible mechanism. First, sUmod and miR-505-5p expression was analyzed within I/R myocardium. Secondly, bioinformatics database and luciferase reporter gene were conducted to predict miR-505-5p's target gene. Finally, function of overexpression of miR-505-5p and sUmod in I/R-mediated myocardial inflammation and apoptosis was analyzed.

## 2. Materials and Methods

### 2.1. Cell Culture

H9C2 cardiomyocytes (ATCC, Manassas, VA, USA) were cultivated within DMEM (Gibco, USA) that contained 1% penicillin/streptomycin (PS) and 10% fetal bovine serum (FBS; Hyclone, Logan, USA) under 37°C and 5% CO_2_ conditions within the humid incubator. Medium change was carried out at 3-day intervals and cell passage was performed after achieving 70-80% density.

### 2.2. Cell Transfection

After reaching 0%-70% density, his work cultured H9C2 cells at 1 day prior to transfection. GenePharma (Shanghai, China) was responsible for preparing miR-505 mimics, miR-505 inhibitor (anti-miR-505), scrambled miRNA (miR-control), and empty control (vector). This work utilized lipofectamine 2000 reagent (Invitrogen, USA) for transfection in line with specific instructions.

### 2.3. Luciferase Reporter Assay

GenePharma (Shanghai, China) was also responsible for preparing mutant (MUT) and wild-type (WT) 3′-UTR in sUmod. Thereafter, MUT or WT 3′-UTR that contained mutations of sUmod binding sites was cloned in downstream luciferase within the empty pMIR vectors (Promega, USA) for producing the recombinant constructs (MUT- or WT-sUmod, separately). Afterwards, by adopting lipofectamine 2000, these constructs were cotransfected into VSMCs with miR-control or miR-505 mimics. In addition, cells were cotransfected with pRL-TK vector (Promega, USA) as well as *Renilla* luciferase gene for control. At 48 h later, dual luciferase assay (Promega, USA) was utilized to measure *Renilla* and firefly luciferase activities in line with specific protocols.

### 2.4. Animals and Ethics

This work obtained forty aged 6-8-week-old male healthy C57BL/6 mice weighing 22-24 g in Beijing Vital River Laboratory Animal Technology Limited Company in China. All mice were then housed in cages at specific temperature and 12 h/12 h light/dark cycle, and they were allowed to drink water and eat chow freely. Each animal experiment was carried out following Guidelines of the Use and Care of Laboratory Animals for Biomedical Research released by National Institutes of Health (No. 85-23, revised 1996). All animals were randomized as the sham and I/R groups. All experimental protocols gained approval from ethics committee.

### 2.5. MIRI Model Establishment

Construction of MIRI model according to previous description [26]. In brief, a 7-0 silk thread was utilized to ligate the left anterior descending branch to establish a mouse myocardial I/R model. Following isoflurane anesthesia, each mouse was intubated and mechanically ventilated, followed by thoracotomy to ligate the left anterior descending branch for a 30 min period. After 3 weeks of reperfusion, the mice were killed for analysis. Except that the left anterior descending branch was not ligated, all steps in the sham group were the same as those in the experimental animals, with the exception of nonligation of left anterior descending branch.

### 2.6. Sampling and Treatment

Following this experiment, mouse serum and heart were collected. Briefly, all animals were anesthetized intraperitoneally using 2% sodium pentobarbital (No.57-33-0, Shanghai Rongbai Biological Technology Company, China) at 1.5 ml/kg. The pleural were opened and the heart was collected. The part of heart tissues in every group was immersed in 10% formalin buffer (No.G2161, Solarbio, China) for pathological examination. The other heart tissues in every group were used for mRNA and protein analysis.

### 2.7. Myocardial Enzyme Detection

The whole blood of mice was placed in the collecting vessel for 30 minutes, 3000 rpm, centrifuged for 10 min, and the serum was taken. The levels of serum BNP, cTnI, CK, and LDH were detected by Hitachi automatic biochemical analyzer 3500.

### 2.8. Histological Analysis

The part of heart tissues in every group were fixed in 10% formaldehyde (No. G2161, Solarbio, China), followed by paraffin embedding with the Leica Microsystem tissue processor (ASP 300S, Germany). Thereafter, 3-5 *μ*m thickness sections were prepared with the Leica Microsystem microtome (Model RM 2265, Germany) to analyze tissue histology by hematoxylin and eosin (H&E) staining.

### 2.9. Quantitative Real-Time PCR (qRT-PCR) Analyses

This work utilized TRIzol reagent (Life Technologies, USA) to extract total tissue RNA, which was then collected to prepare the first-strand cDNA using SuperScript II RT Kit (Invitrogen, USA) Reverse Transcription of miR-505-5p was done using miScript Reverse Transcription Kit (Qiagen, Germany) .. Subsequently, SYBR Green Real-time PCR kit was used to measure miR-505-5p level using the ABI Prism7500 sequence detection system (Applied Biosystems, USA). Shengke Company (Guangzhou, China) was responsible for preparing primers used in qRT-PCR. miR-505-5p, forward (F): 5′-GTAATCGGGAGCCAGGAAGT-3′, reverse (R): 5′-GTGTCGTGGAGTCGGCAAT-3′; U6 (F): 5′-ATTGGAACGATACAGAG AAGATT-3′; (R): 5′-AGGAACGCTTCACGAATTTG-3′. PCR program is 5 min under 95°C; 15 s under 95°C and 60 s under 60°C for altogether 40 cycles. 2^−ΔΔCt^ was utilized to determine fold change (FC) of miR-505-5p by normalizing to GAPDH.

### 2.10. Western Blot (WB) Assay

This work utilized tissue protein extraction kit (No. FD0889, Hangzhou Fude Biological Technology Company, China) to extract total tissue proteins. Thereafter, the BCA protein assay kit (No. FD2001, Hangzhou Fude Biological Technology Company, China) was utilized to measure protein content. Subsequently, proteins were separated by 10%SDS-PAGE after 5 min sample boiling and denaturation, followed by transfer onto 5% defatted milk PBST buffer for a 1 h period under ambient temperature. After washing by PBST thrice, membranes were incubated with primary antibodies shown in Table 1 under 4°C overnight. After further washing by PBST thrice, membranes were incubated with appropriate secondary antibody for 1.5 h. The Clarity™ Western ECL Substrate (No.170-5061, Bio-Rad Laboratories, USA) was utilized for protein visualization using the Tanon 5200 chemiluminescence image analysis system (Tanon Science and Technology Co., Ltd., Shanghai, China). The absorbance (OD) ratio of target protein to GAPDH was used as the relative expression level of the target protein.

### 2.11. Statistical Analyses

Results were represented by mean ± SD and examined by SPSS20.0 (Chicago, IL, USA). Student's *t*-test was conducted to compare 2 groups, while one-way ANOVA combined with Tukey's test was adopted to compare several groups. *P* < 0.05 stood for statistical significance.

## 3. Results

### 3.1. sUmod Showed High Expression within MIRI

**Figure 1:**
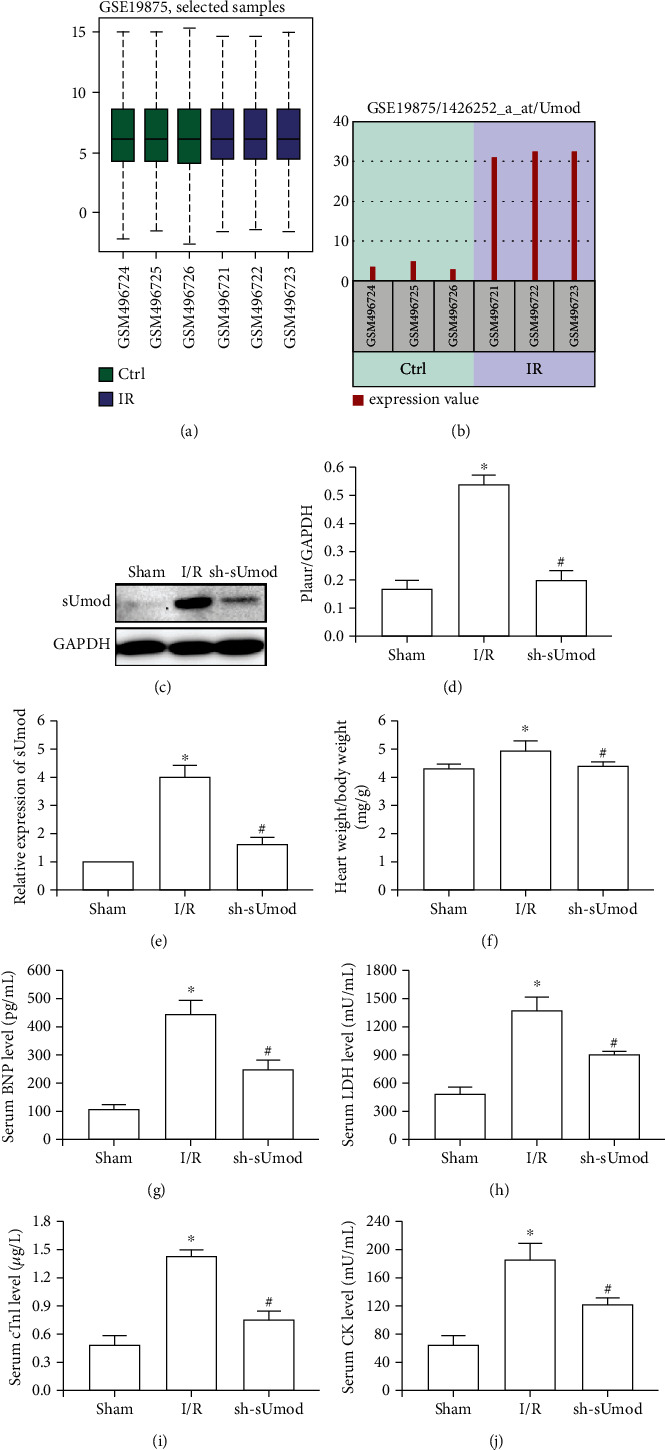
sUmod was highly expressed in myocardial I/R injury. (a and b) According to the target prediction of bioinformatics database, sUmod was highly expressed in myocardial I/R injury. (c and d) Western blot analysis showed that sUmod was also highly expressed in myocardial I/R injury. (e) qRT-PCR was used to analyze the expression levels of sUmod mRNA in each group.(f)Heart weight/body weight ratio in each group. (g–j) The expression levels of serum myocardial enzymes BNP (g), LDH (h), cTnl (i), and CK (j). ^∗^*P* < 0.05 vs. Sham; ^#^*P* < 0.05 vs. I/R.

As observed from Figure 1(a), the symmetrical gene expression distribution within diverse samples were consistent with others, which indicated the absence of interference within samples and valid cross-comparison. Through analyzing GEO2R in GEO database, the data showed that sUmod was abnormally highly expressed in MIRI relative to sham group (Figure 1(b)). As revealed by western blot and qRT-PCR, sUmod showed abnormally high expression within MIRI group (Figures 1(c)–1(e)). Moreover, relative to I/R group, sUmod silencing remarkably declined the ratio of heart weight to body weight (BW) (Figure 1(f)), and reduced serum expression of BNP, LDH, cTnl, and CK (Figures 1(g)–1(j)).

### 3.2. sUmod Silencing Ameliorated I/R-Mediated Myocardial Apoptosis and Inflammation

**Figure 2:**
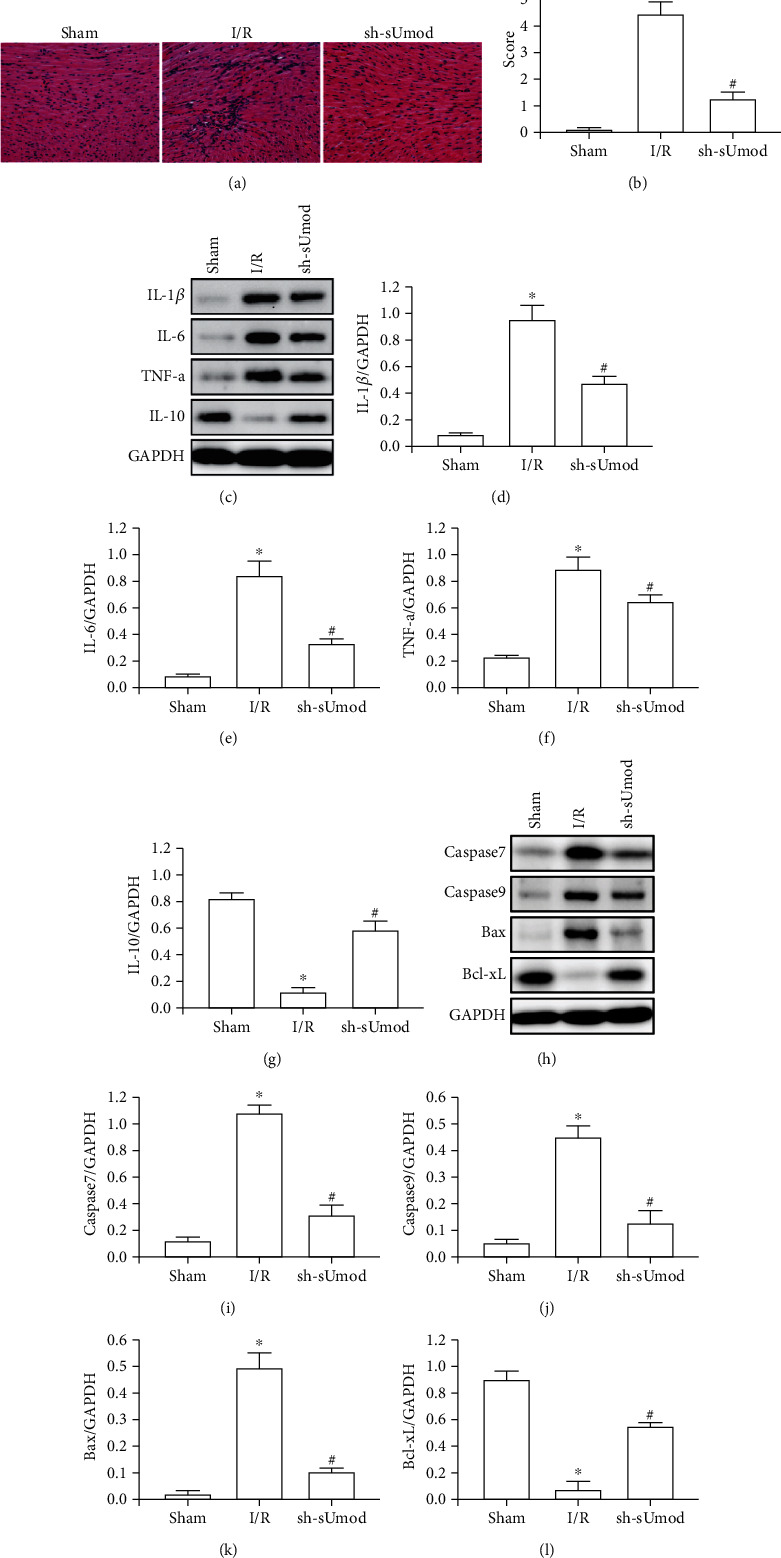
sUmod silencing ameliorates I/R-induced myocardial inflammation and apoptosis. (a and b) Pathological changes and injury score in myocardial I/R injury (HE staining, bar = 100 *μ*m). (c–g) Western blot was used to quantitatively analyze the expression of IL-1*β*, IL-6, TNF-*α*, and IL-10 in myocardial I/R injury. (h–l) Western blot was used to quantitatively analyze the expression of caspase-7, caspase-9, Bax, and Bcl-xL in myocardial I/R injury. ^∗^*P* < 0.05 vs. Sham; ^#^*P* < 0.05 vs. I/R.

HE staining and WB assays were conducted to detect effects on I/R-mediated myocardial apoptosis and inflammation. Our data showed that I/R led to myocardial degeneration/necrosis, inflammatory cells infiltration, whereas sUmod silencing effectively improved MIRI score (Figures 2(a) and 2(b)), decreased TNF-*α*, IL-1*β*, IL-6 levels, but increased IL-10 expression (Figures 2(c)–2(g)). Moreover, relative to sham group, I/R dramatically enhanced caspase7, caspase9 and Bax expression, and decreased Bcl-xL expression, whereas sUmod silencing effectively decreased caspase7, caspase9 and Bax levels, and elevated Bcl-xL level (Figures 2(h)–2(l)).

### 3.3. sUmod Was miR-505-5p's Direct Target Gene

**Figure 3:**
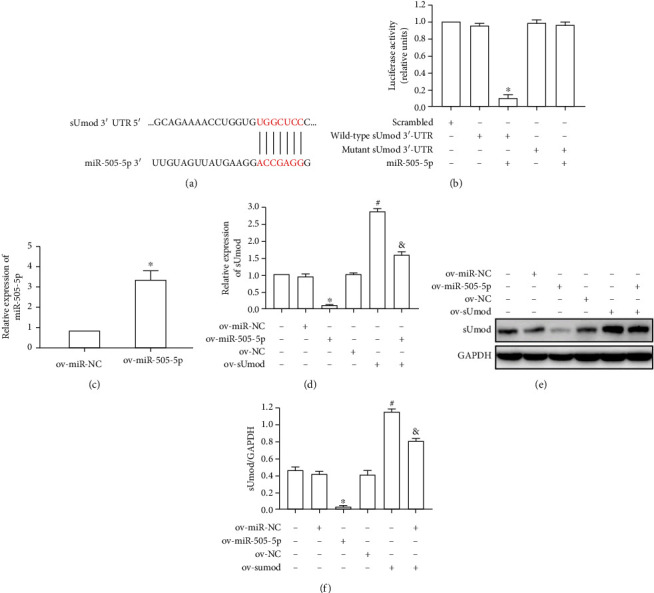
sUmod was a direct target gene of miR-505-5p. (a) The putative miR-505-5p-binding sequence in the 3′-UTR of sUmod mRNA. (b) Luciferase reporter assay of sUmod-WT and sUmod-Mut constructs in H9C2 cells transfected with ov-miR-NC or ov-miR-505-5p. (c) qRT-PCR were used to analyze the expression of miR-505-5p in H9C2 cells transfected with ov-miR-NC or ov-miR-505-5p. (d–f) Western blot and qRT-PCR were used to analyze the effects of ov-miR-505-5p and ov-sUmod on sUmod protein and mRNA expression.^∗^*P* < 0.05 vs. ov-miR-NC; ^#^*P* < 0.05 vs. ov-NC; ^&^*P* < 0.05 vs. ov-miR-505-5p and ov-sUmod.

Target was predicted by TargetScan bioinformatics database, and the potential regulatory factor miR-505-5p of sUmod was obtained (Figure 3(a)). According to luciferase reporter gene assay, miR-505-5p overexpression in cardiomyocytes H9C2 significantly suppressed luciferase activities in wild-type-sUmod group (Figure 3(b)). miR-505-5p plasmid transfection significantly upregulated miR-505-5p level within H9C2 cells (Figure 3(c)). Moreover, miR-505-5p upregulation remarkably inhibited sUmod level in H9C2 cells, and sUmod overexpression offset the effect of miR-505-5p upregulation (Figures, 3(d)–3(f)), indicating that miR-505-5p targeted regulation of sUmod expression.

### 3.4. miR-505-5p Overexpression Attenuated I/R-Mediated MIRI

**Figure 4:**
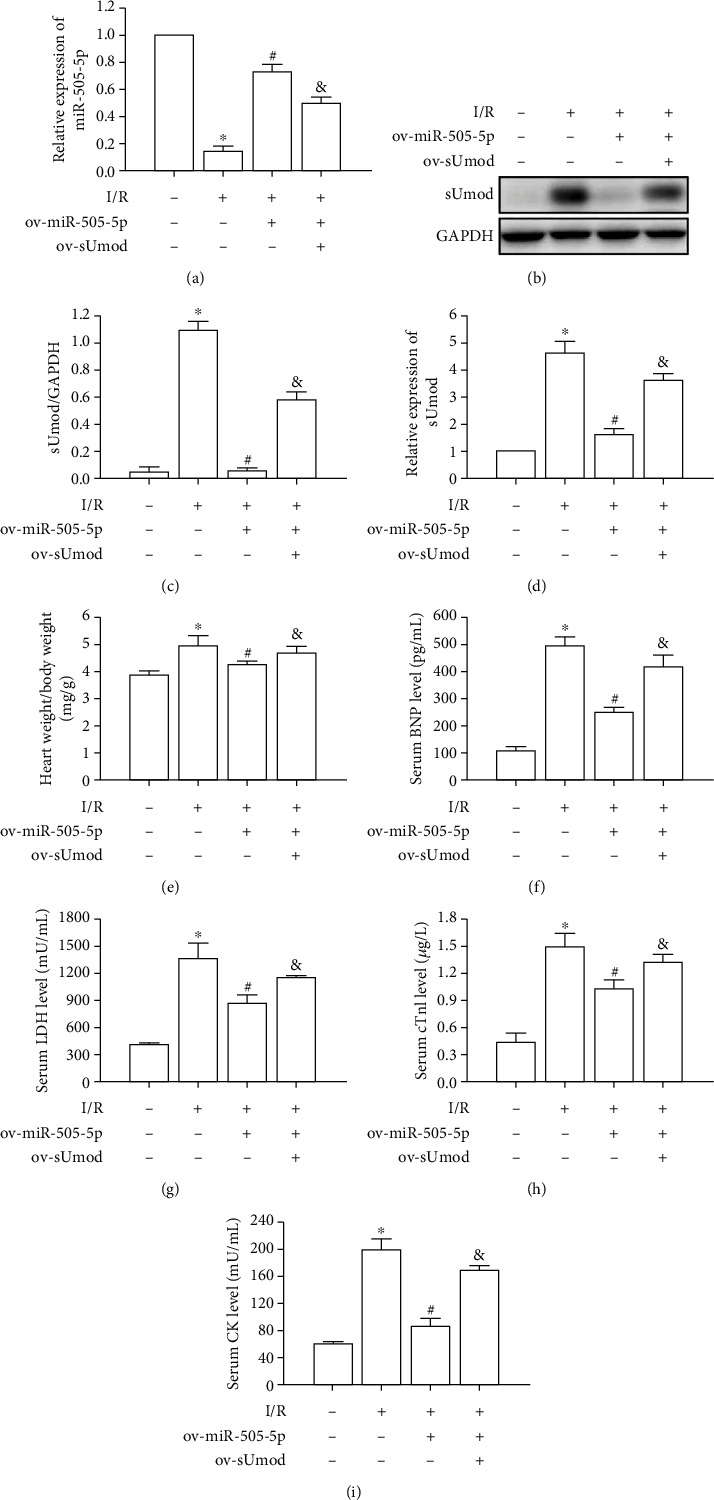
miR-505-5p overexpression attenuated I/R-induced myocardial injury. (a) The expression of miR-505-5p in I/R myocardium with ov-miR-505-5p and ov-sUmod was analyzed by qRT-PCR.(b–d) Western blot and qRT-PCR were used to analyze the effects of ov-miR-505-5p and ov-sUmod on sUmod protein and mRNA expression in myocardial I/R injury. (e) The effect of ov-miR-505-5p and ov-sUmod on heart weight/body weight ratio. (f and i) The effects of ov-miR-505-5p and ov-sUmod on serum cardiac enzymes BNP (f), LDH (g), cTnl (h), and CK (i). ^∗^*P* < 0.05 vs. Sham; ^#^*P* < 0.05 vs. I/R; ^&^*P* < 0.05 vs. ov- miR-505-5p.

miR-505-5p showed low expression within MIRI, and miR-505-5p upregulation significantly upregulated miR-505-5p level, and was reversed by overexpression of sUmod (Figure 4(a)). According to qRT-PCR and WB assays, miR-505-5p upregulation inhibited sUmod induced by MIRI. Meanwhile, sUmod upregulation remarkably offset decrease of sUmod expression induced by miR-505-5p overexpression (Figures 4(b)–4(d)). Moreover, relative to I/R group, miR-505-5p upregulation significantly inhibited the heart weight/body weight ratio, and reduced serum BNP, LDH, cTnl, and CK expression, whereas the overexpression of sUmod counteracted miR-505-5p overexpression's impact on heart weight/body weight ratio, serum BNP, LDH, cTnl, and CK (Figures 4(e)–4(i)).

### 3.5. miR-505-5p Overexpression Improved I/R-Induced Myocardial Inflammation and Apoptosis

**Figure 5:**
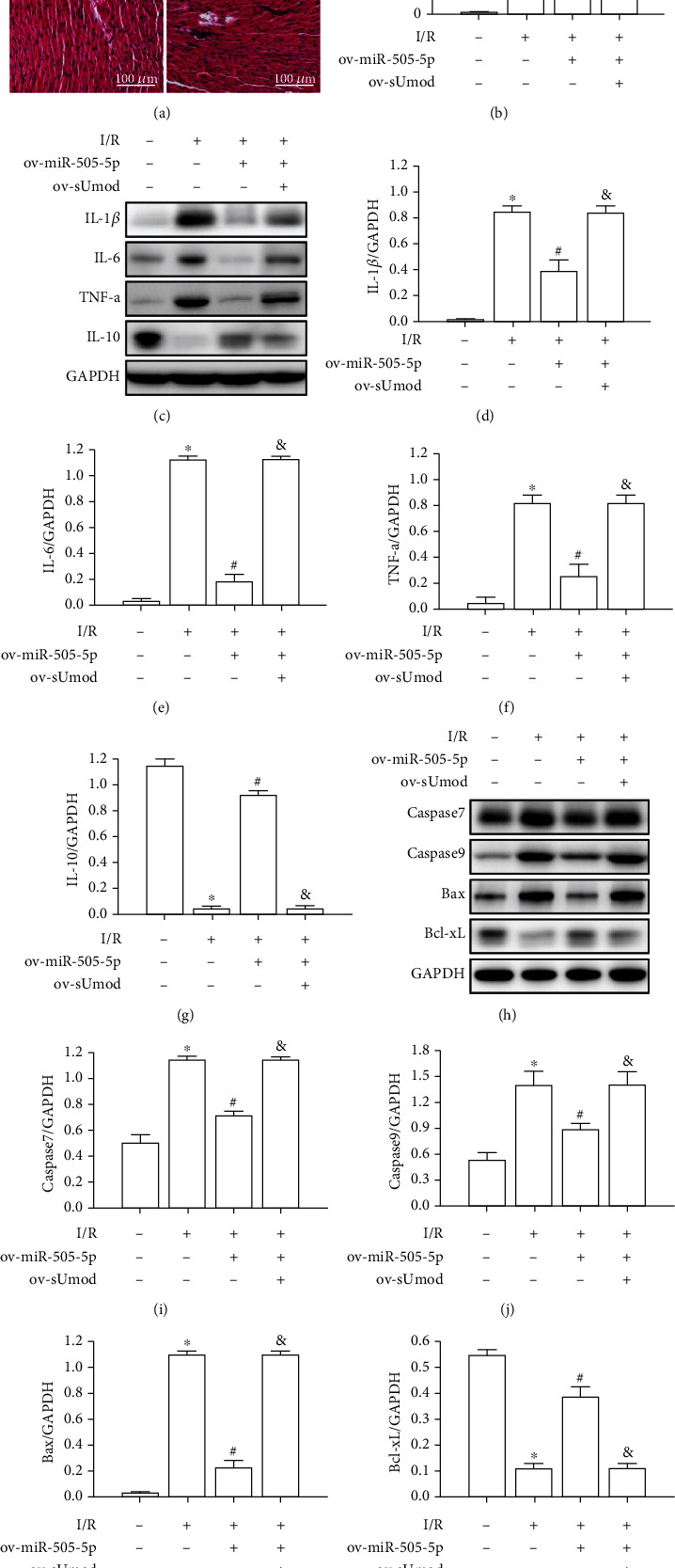
miR-505-5p overexpression attenuated I/R-induced myocardial inflammation and apoptosis. (a and b) The Effects of ov-miR-505-5p and ov-sUmod on myocardial pathological changes and injury score after myocardial I/R injury (HE staining, bar = 100 *μ*m). (c–g) Quantitative analysis of IL-1*β*, IL-6, TNF-*α*, and IL-10 in myocardial I/R injury with ov-miR-505-5p and ov-sUmod by western blot analysis. (h–l) Quantitative analysis of caspase 7, caspase 9, Bax, and Bcl-xL in myocardial I/R injury with ov-miR-505-5p and ov-sUmod by western blot analysis. ^∗^*P* < 0.05 vs. Sham; ^#^*P* < 0.05 vs. I/R; ^&^*P* < 0.05 vs. ov-miR-505-5p.

Our data showed that I/R caused cardiomyocyte degeneration and necrosis, inflammatory cell infiltration, miR-505-5p overexpression effectively improved MIRI score, whereas sUmod upregulation significantly counteracted miR-505-5p overexpression on MIRI score (Figures 5(a) and 5(b)). Moreover, relative to sham group, I/R-mediated increases in TNF-*α*, IL-1*β* and IL-6 levels, whereas declined IL-10 expression, and miR-505-5p overexpression effectively decreased TNF-*α*, IL-1*β* and IL-6 levels, but increased IL-10 expression, whereas sUmod upregulation remarkably offset miR-505-5p overexpression's impact on myocardial I/R-induced inflammation (Figures 5(c)–5(g)). Furthermore, I/R remarkably upregulated caspase7, caspase9, and Bax levels, and declined Bcl-xL expression, and miR-505-5p upregulation effectively suppressed the expression of caspase7, caspase9 and Bax, and increased Bcl-xL expression, whereas sUmod upregulation remarkably counteracted miR-505-5p overexpression's impact on myocardial I/R-induced apoptosis (Figures 5(h) –5(l)).

## 4. Discussion

It has been found that miR-505-5p is closely related to cardiovascular metabolic risk factors [27]. In this study, our data showed that sUmod was miR-505-5p's direct target gene. Meanwhile, miR-505-5p and sUmod showed high expression within MIRI, which were associated with increased serum myocardial enzyme level. miR-505-5p or sUmod silencing effectively improved MIRI score and inhibit myocardial apoptosis and inflammation. sUmod upregulation can counteract the miR-505-5p overexpression's effect on inhibiting MIRI-mediated apoptosis and inflammation. Based on the results, miR-505-5p improve myocardial I/R-mediated apoptosis and inflammation through inhibiting sUmod.

Up to date, according to the report, I/R leads to inflammation, apoptosis, iron death and fibrosis, and even cardiac arrest, and inflammation and apoptosis have an indispensable effect on MIRI genesis [28]. The inflammatory response is supported by macrophages in the heart and white blood cells in circulation, which can easily enter the stroma through damaged vascular endothelial cells. Moreover, if the ischemic period is long enough, the death process of parenchyma cells and cardiomyocytes is activated, mainly because of cell necrosis, followed by apoptosis and autophagy [29]. The role of leukocytes and mitochondria during MIRI plays a key effect on the I/R-induced inflammatory mechanism. Macrophages can show different phenotypes according to the state and phase of inflammation. M1 macrophage represents proinflammatory characteristics, while M2 macrophage can promote wound healing, and downregulate several cytokines. M1 macrophages activate NF-*κ*B signaling pathway by binding to MyD88, thus transcribing TNF-*α*, IL-1*β*, IL-6, and IL-12 expression. Macrophages polarize M2 phenotypes by stimulating glucocorticoid receptors, IL-10 receptors, IL-3, and IL-14 distributed within B cells, T cells, macrophages, and mast cells. Our data showed that sUmod showed high expression within MIRI, which was associated with increased serum myocardial enzyme level. Meanwhile, sUmod silencing can effectively promote MIRI score, inhibit myocardial inflammation and apoptosis. sUmod was reported to interact with myoloid dendritic cells (DCs), monocytes, and neutrophils through toll-like receptor 4 (TLR4), and has an effect on the regulation of inflammation as well as innate immunity [30], and the immunosuppressive effect of sUmod was achieved by binding to TNF-*α* and interleukin-1 [31]. In addition, sUmod stimulated extrarenal tissue, and caused strong inflammatory response, which was characterized by obviously recruiting inflammatory monocytes and neutrophils [32]. sUmod induced the secretion of TNF-*α* and the expression of tissue factors in human monocytes, triggered DC maturation through activating NF-*κ*B and TLR4, and activated protease release, respiratory burst, degranulation, and phagocytosis of neutrophils [33]. Intravenous injection of sUmod caused systemic inflammation, and the proinflammatory effect of sUmod was also described [34]. In contrast, sUmod limited inflammation by inhibiting proinflammatory cytokines and chemokines, which negatively regulated granulocyte production and neutrophil homeostasis in the system [35, 36]. Furthermore, sUmod was also reported to inhibit the activation of nonselective calcium channel TRPM2 in mice and reduce the increase of reactive oxygen species in kidney and systemic acute renal injury [37]. sUmod enhanced the expression of CXCL8, inhibited the expression of human granulocyte CD62L [38], stimulated NLRP3 inflammatory bodies within human monocytes, causing IL-1*β* production and cell apoptosis [39]. The results suggest that sUmod can improve myocardial I/R-mediated apoptosis and inflammation.

miRNAs were related to MIRI through altering apoptosis, inflammation, and fibrosis. According to our results, miR-505-5p was expressed highly during myocardial I/R injury, and altered serum myocardial enzyme expression. miR-505-5p silencing can effectively improve MIRI score, apoptosis, and inflammation. miR-505-5p shows low expression in breast cancer and is one of the most valuable biomarkers to identify breast cancer [40]. miR-505-5p promoted lung cancer cell growth via TP53AIP1 and plays the role of oncogenes [24]. Furthermore, miR-505-5p level in osteosarcoma significantly elevated, whereas inhibiting miR-505-5p upregulated RASSF8, thus inhibiting osteosarcoma cell proliferation and promote their apoptosis [41]. More importantly, miR-505-5p targeted SRSF1 (splicing factor 1 rich in serine/arginine), while SRSF1 thus regulated enoglin, tissue factors and vascular endothelial growth factor A (VEGFA), and controled the endothelial cell molecular aging process, causing age-related vascular disease [27, 42]. Besides, miR-505-3p is another member of the miR-505 family, whose expression also increased in osteoarthritis, whereas overexpression of circFAM160A2 improved mitochondrial dysfunction and chondrocyte apoptosis through inhibiting miR-505-3p. These results suggest that circFAM160A2 can decrease chondrocyte apoptosis in osteoarthritis via miR-505-3p [43]. Collectively, miR-505-5p improved myocardial I/R-mediated inflammation and apoptosis.

miR-505 is a critical regulating factor for chronic inflammation in mammals [44]. Our data demonstrated that sUmod is miR-505-5p's direct downstream target, and sUmod overexpression counteract miR-505-5p konckdown's impact on I/R-induced myocardial apoptosis and inflammation. As discovered in a similar study, miR-505 level within lipopolysaccharide-induced endometritis dramatically decreases. Overexpression of miR-505 can significantly decrease TNF-*α*, IL-1*β*, IL-6 levels, whereas inhibition of miR-505 can get the opposite result. Moreover, double luciferase analysis confirmed that miR-505 targets noncoding region in HMGB13′ and regulates NF- *κ*B signaling activation mediated by lipopolysaccharide, thus reducing the production of proinflammatory cytokines [45]. In addition, oxidized low density lipoprotein was reported to induce miR-505expression, and inhibit sirt3 expression through activating NF-*κ*B pathway, which leads to neutrophils releasing reactive oxygen species (ROS) [46]. Such results help to understand miR-505-5p for its effect against apoptosis and inflammation during MIRI, and the miR-505-5p/sUmod axis may help to limit myocardial ischemic injury.

In short, this study identifies sUmod as miR-505-5p's direct downstream target gene. miR-505-5p overexpression can effectively improve I/R-mediated myocardial apoptosis and inflammation by targeting inhibition of sUmod expression.

## Figures and Tables

**Table 1:** The appropriate primary antibody.

Antibody	Dilution	Product number	Product company
THP	1 : 1500	Sc-20361	Santa Cruz Biotechnology, USA
IL-1*β*	1 : 1000	Sc-7884	Santa Cruz Biotechnology, USA
IL-6	1 : 1000	Sc-73318	Santa Cruz Biotechnology, USA
IL-10	1 : 1000	ab9969	Abcam, UK
TNF-*α*	1 : 500	ab8348	Abcam, UK
Caspase7	1 : 100	Sc-56063	Santa Cruz Biotechnology, USA
Caspase9	1 : 1000	Sc-56076	Santa Cruz Biotechnology, USA
Bax	1 : 2000	Sc-526	Santa Cruz Biotechnology, USA
Bcl-xL	1 : 1000	ab32370	Abcam, UK
GAPDH	1 : 5000	Sc-32233	Santa Cruz Biotechnology, USA
Goat anti-mouse secondary antibody	1 : 4000	ab6721	Abcam, UK
Goat anti-rat secondary antibody	1 : 2000	ab7097	Abcam, UK

## Data Availability

Data will be available from the corresponding authors under request.

## References

[1] Chen M., Li X., Yang H., Tang J., Zhou S. (2020). Hype or hope: vagus nerve stimulation against acute myocardial ischemia-reperfusion injury. *Trends in Cardiovascular Medicine*.

[2] Satomi S., Morio A., Miyoshi H. (2020). Branched-chain amino acids-induced cardiac protection against ischemia/reperfusion injury. *Life Sciences*.

[3] Xin C., Zhang Z., Gao G. (2020). Irisin attenuates myocardial ischemia/reperfusion injury and improves mitochondrial function through AMPK pathway in diabetic mice. *Frontiers in Pharmacology*.

[4] Davidson S. M., Adameová A., Barile L. (2020). Mitochondrial and mitochondrial-independent pathways of myocardial cell death during ischaemia and reperfusion injury. *Journal of Cellular and Molecular Medicine*.

[5] Yu L.-M., Dong X., Xue X.-D. (2021). Melatonin attenuates diabetic cardiomyopathy and reduces myocardial vulnerability to ischemia-reperfusion injury by improving mitochondrial quality control: role of SIRT6. *Journal of Pineal Research*.

[6] Ahmed S., Ahmed N., Rungatscher A. (2020). Cocoa flavonoids reduce inflammation and oxidative stress in a myocardial ischemia-reperfusion experimental model. *Antioxidants (Basel)*.

[7] Chien C.-Y., Wen T.-J., Cheng Y.-H., Tsai Y.-T., Chiang C.-Y., Chien C.-T. (2020). Diabetes upregulates oxidative stress and downregulates cardiac protection to exacerbate myocardial ischemia/reperfusion injury in rats. *Antioxidants (Basel)*.

[8] Wang J., Toan S., Li R., Zhou H. (2020). Melatonin fine-tunes intracellular calcium signals and eliminates myocardial damage through the IP3R/MCU pathways in cardiorenal syndrome type 3. *Biochemical Pharmacology*.

[9] Peng K., Liu H., Yan B. (2021). Inhibition of cathepsin S attenuates myocardial ischemia/reperfusion injury by suppressing inflammation and apoptosis. *Journal of Cellular Physiology*.

[10] Köhler D., Granja T., Volz J. (2020). Red blood cell-derived semaphorin 7A promotes thrombo-inflammation in myocardial ischemia-reperfusion injury through platelet GPIb. *Nature Communications*.

[11] Pei Y.-H., Chen J., Wu X. (2020). LncRNA PEAMIR inhibits apoptosis and inflammatory response in PM2.5 exposure aggravated myocardial ischemia/reperfusion injury as a competing endogenous RNA of miR-29b-3p. *Nanotoxicology*.

[12] Devuyst O., Olinger E., Rampoldi L. (2017). Uromodulin: from physiology to rare and complex kidney disorders. *Nature Reviews. Nephrology*.

[13] Delgado G. E., Kleber M. E., Scharnagl H., Krämer B. K., März W., Scherberich J. E. (2017). Serum uromodulin and mortality risk in patients undergoing coronary angiography. *Journal of the American Society of Nephrology*.

[14] Then C., Then H., Meisinger C. (2019). Serum uromodulin is associated with but does not predict type 2 diabetes in elderly KORA F4/FF4 study participants. *The Journal of Clinical Endocrinology and Metabolism*.

[15] Leiherer A., Muendlein A., Saely C. H. (2017). Serum uromodulin is associated with impaired glucose metabolism. *Medicine (Baltimore)*.

[16] Tsai C. Y., Wu T. H., Yu C. L., Lu J. Y., Tsai Y. Y. (2000). Increased excretions of beta2-microglobulin, IL-6, and IL-8 and decreased excretion of Tamm-Horsfall glycoprotein in urine of patients with active lupus nephritis. *Nephron*.

[17] Micanovic R., Khan S., Janosevic D. (2018). Tamm-Horsfall protein regulates mononuclear phagocytes in the kidney. *Journal of the American Society of Nephrology*.

[18] Kozomara A., Griffiths-Jones S. (2014). miRBase: annotating high confidence microRNAs using deep sequencing data. *Nucleic Acids Research*.

[19] Zhang C., Yang X., Fu C., Liu X. (2018). Combination with TMZ and miR-505 inhibits the development of glioblastoma by regulating the WNT7B/WNT/*β*-catenin signaling pathway. *Gene*.

[20] Lu L., Zhang D., Xu Y., Bai G., Lv Y., Liang J. (2018). miR-505 enhances doxorubicin-induced cytotoxicity in hepatocellular carcinoma through repressing the Akt pathway by directly targeting HMGB1. *Biomedicine & Pharmacotherapy*.

[21] Song C.-J., Chen H., Chen L.-Z., Ru G.-M., Guo J.-J., Ding Q.-N. (2018). The potential of microRNAs as human prostate cancer biomarkers: a meta-analysis of related studies. *Journal of Cellular Biochemistry*.

[22] Guo Y., Xu X., Tang T. (2022). miR-505 inhibits replication of Borna disease virus 1 via inhibition of HMGB1-mediated autophagy. *The Journal of General Virology*.

[23] Zhao S., Lu L., Liu Q. (2019). MiR-505 promotes M2 polarization in choroidal neovascularization model mice by targeting transmembrane protein 229B. *Scandinavian Journal of Immunology*.

[24] Fang H., Liu Y., He Y. (2019). Extracellular vesicle-delivered miR-505-5p, as a diagnostic biomarker of early lung adenocarcinoma, inhibits cell apoptosis by targeting TP53AIP1. *International Journal of Oncology*.

[25] Kapora E., Feng S., Liu W., Sakhautdinova I., Gao B., Tan W. (2019). MicroRNA-505-5p functions as a tumor suppressor by targeting cyclin-dependent kinase 5 in cervical cancer. *Bioscience Reports*.

[26] Gao R., Wang L., Bei Y. (2021). Long noncoding RNA cardiac physiological hypertrophy-associated regulator induces cardiac physiological hypertrophy and promotes functional recovery after myocardial ischemia-reperfusion injury. *Circulation*.

[27] McManus D. D., Rong J., Huan T. (2017). Messenger RNA and MicroRNA transcriptomic signatures of cardiometabolic risk factors. *BMC Genomics*.

[28] Lv X., Lu P., Hu Y., Xu T. (2020). miR-346 inhibited apoptosis against myocardial ischemia-reperfusion injury via targeting Bax in rats. *Drug Design, Development and Therapy*.

[29] Algoet M., Janssens S., Himmelreich U. (2022). Myocardial ischemia-reperfusion injury and the influence of inflammation. *Trends in Cardiovascular Medicine*.

[30] Padmanabhan S., Graham L., Ferreri N. R., Graham D., McBride M., Dominiczak A. F. (2014). Uromodulin, an emerging novel pathway for blood pressure regulation and hypertension. *Hypertension*.

[31] Kirkham M., Fujita A., Chadda R. (2005). Ultrastructural identification of uncoated caveolin-independent early endocytic vehicles. *The Journal of Cell Biology*.

[32] Immler R., Lange-Sperandio B., Steffen T. (2020). Extratubular polymerized uromodulin induces leukocyte recruitment and inflammation in vivo. *Frontiers in Immunology*.

[33] Siao S.-C., Li K.-J., Hsieh S.-C. (2011). Tamm-Horsfall glycoprotein enhances PMN phagocytosis by binding to cell surface-expressed lactoferrin and cathepsin G that activates MAP kinase pathway. *Molecules*.

[34] Säemann M. D., Weichhart T., Zeyda M. (2005). Tamm-Horsfall glycoprotein links innate immune cell activation with adaptive immunity via a toll-like receptor-4-dependent mechanism. *The Journal of Clinical Investigation*.

[35] El-Achkar T. M., McCracken R., Liu Y. (2013). Tamm-Horsfall protein translocates to the basolateral domain of thick ascending limbs, interstitium, and circulation during recovery from acute kidney injury. *American Journal of Physiology. Renal Physiology*.

[36] Micanovic R., Chitteti B. R., Dagher P. C. (2015). Tamm-Horsfall protein regulates granulopoiesis and systemic neutrophil homeostasis. *Journal of the American Society of Nephrology*.

[37] Then C., Then H. L., Lechner A. (2020). Serum uromodulin and risk for cardiovascular morbidity and mortality in the community-based KORA F4 study. *Atherosclerosis*.

[38] Kreft B., Jabs W. J., Laskay T. (2002). Polarized expression of Tamm-Horsfall protein by renal tubular epithelial cells activates human granulocytes. *Infection and Immunity*.

[39] Darisipudi M. N., Thomasova D., Mulay S. R. (2012). Uromodulin triggers IL-1*β*-dependent innate immunity via the NLRP3 inflammasome. *Journal of the American Society of Nephrology*.

[40] Matamala N., Vargas M. T., González-Cámpora R. (2015). Tumor microRNA expression profiling identifies circulating microRNAs for early breast cancer detection. *Clinical Chemistry*.

[41] Wang T., Zhang H., Wang H., Chang C., Huang F., Zhang L. (2021). MiR-505-5p inhibits proliferation and promotes apoptosis of osteosarcoma cells via regulating RASSF8 expression. *Journal of BUON*.

[42] Davies R. W., Wells G. A., Stewart A. F. R. (2012). A genome-wide association study for coronary artery disease identifies a novel susceptibility locus in the major histocompatibility complex. *Circulation. Cardiovascular Genetics*.

[43] Bao J., Lin C., Zhou X. (2021). circFAM160A2 promotes mitochondrial stabilization and apoptosis reduction in osteoarthritis chondrocytes by targeting miR-505-3p and SIRT3. *Oxidative Medicine and Cellular Longevity*.

[44] Escate R., Mata P., Cepeda J. M., Padró T., Badimon L. (2018). miR-505-3p controls chemokine receptor up-regulation in macrophages: role in familial hypercholesterolemia. *The FASEB Journal*.

[45] Liu J., Guo S., Zhang T. (2020). MiR-505 as an anti-inflammatory regulator suppresses HMGB1/NF-*κ*B pathway in lipopolysaccharide-mediated endometritis by targeting HMGB1. *International Immunopharmacology*.

[46] Chen L., Hu L., Li Q., Ma J., Li H. (2019). Exosome-encapsulated miR-505 from ox-LDL-treated vascular endothelial cells aggravates atherosclerosis by inducing NET formation. *Acta Biochimica et Biophysica Sinica*.

